# Systematic identification of key extracellular proteins as the potential biomarkers in lupus nephritis

**DOI:** 10.3389/fimmu.2022.915784

**Published:** 2022-07-28

**Authors:** Xue Zhou, Yuefeng Zhang, Ning Wang

**Affiliations:** ^1^ Department of Nephrology, Tianjin Haihe Hospital, Tianjin, China; ^2^ Haihe Hospital, Tianjin University, Tianjin, China; ^3^ Haihe Clinical School, Tianjin Medical University, Tianjin, China; ^4^ Medical Department, The Third Central Hospital of Tianjin, Tianjin, China

**Keywords:** lupus nephritis, diagnosis, biomarker, bioinformatics analysis, extracellular proteins

## Abstract

**Background:**

Lupus nephritis (LN) is the most common and severe clinical manifestation of systemic lupus erythematosus (SLE) with considerable morbidity/mortality and limited treatment options. Since kidney biopsy is a relative hysteretic indicator, it is indispensable to investigate potential biomarkers for early diagnosis and predicting clinical outcomes of LN patients. Extracellular proteins may become the promising biomarkers by the secretion into body fluid. Our study linked extracellular proteins with lupus nephritis to identify the emerging biomarkers.

**Methods:**

The expression profiling data were acquired from the Gene Expression Omnibus (GEO) database. Meanwhile, the two gene lists encoding extracellular proteins were collected from the Human Protein Atlas (HPA) and UniProt database. Subsequently, the extracellular protein-differentially expressed genes (EP-DEGs) were screened out, and the key EP-DEGs were determined by MCODE, MCC, and Degree methods *via* the protein–protein interaction (PPI) network. The expression level, immune characteristics, and diagnostic value of these candidate biomarkers were investigated. Finally, the Nephroseq V5 tool was applied to evaluate the clinical significance of the key EP-DEGs.

**Results:**

A total of 164 DEGs were acquired by comparing LN samples with healthy controls based on GSE32591 datasets. Then, 38 EP-DEGs were screened out through the intersection between DEGs and extracellular protein gene lists. Function enrichment analysis indicated that these EP-DEGs might participate in immune response and constitute the extracellular matrix. Four key EP-DEGs (LUM, TGFBI, COL1A2, and POSTN) were eventually identified as candidate biomarkers, and they were all overexpressed in LN samples. Except that LUM expression was negatively correlated with most of the immune regulatory genes, there was a positive correlation between the remaining three biomarkers and the immune regulatory genes. In addition, these biomarkers had high diagnostic value, especially the AUC value of the LUM–TGFBI combination which reached almost 1 (AUC = 0.973), demonstrating high accuracy in distinguishing LN from controls. Finally, we found a meaningful correlation of these biomarkers with sex, WHO class, and renal function such as glomerular filtration rate (GFR), serum creatinine level, and proteinuria.

**Conclusion:**

In summary, our study comprehensively identified four key EP-DEGs exerting a vital role in LN diagnosis and pathogenesis and serving as promising therapeutic targets.

## Introduction

Lupus nephritis (LN) is the most common and severe clinical manifestation of systemic lupus erythematosus (SLE) leading to irreversible renal impairment with considerable morbidity/mortality and limited treatment options (1–3). It is estimated that 10%–20% of LN patients eventually develop end-stage renal disease (ESRD) (4, 5). The pathogenesis of LN remains poorly understood due to immune and non-immune mechanisms (6). It is triggered by the deposition of nucleic acid-containing substances in the glomerulus leading to the activation of complement and renal interstitial cells and the recruitment of inflammatory cells (7). The salient features of LN include highly correlated systemic inflammation, the deposition of immune complexes, the activation of inflammasome, type I interferon-mediated endothelial dysfunction (8, 9), complement-mediated injury (10), and thrombophilia. When a patient develops hematuria, proteinuria, or renal function abnormality, the definitive renal biopsy is required for LN diagnosis.

Currently, none of the new biomarkers being superior to the estimated glomerular filtration rate (GFR) or proteinuria have been applied in clinical practice. Meanwhile, an early diagnosis of LN is difficult, and renal biopsy may not predict the prognosis of LN. Development of suitable biomarkers with high sensitivity and specificity has provided significant insights regarding diagnosis, prognosis, treatment selection, and personalized treatment strategies for patients. Consequently, it is desperately needed to explore the reliable biomarkers contributing to accurately evaluating disease status and guiding the selection of precision therapies for LN patients. Substantial evidence exists to support the changes of specific extracellular proteins in various body fluids that can be used as potential predictors for disease development and progression. Some small molecules can be recognized in human body fluids and tissues, which may be considered as the potential biomarkers or therapeutic targets in LN patients (11–15). Consequently, it has created our immense interest in finding potential biomarkers that can provide meaningful information in diagnosis, prognosis, and clinical treatment for LN patients.

Some studies have found that extracellular proteins can be considered as novel biomarkers and therapeutic targets in many diseases, including renal dysfunction (16), renal damage (17), hyperthyroid heart disease (18), and pulmonary arterial hypertension (19). In addition, extracellular proteins serve as biomarkers for diagnosis and the development and progression in multiple cancer types, involving lung cancer (20), prostate cancer (21), melanoma (22), and ovarian cancer (23). Extracellular proteins serving as potential biomarkers have important biological function and participate in the pathogenesis of SLE and LN. Recent research suggests that extracellular HMGB1 has been defined as an important biomarker and new therapeutic target in SLE (24). Interferon (IFN) α, a key cytokine, deriving from selectively activated neutrophils exerts a crucial role in the pathogenesis of SLE (25). Further studies in LN pathogenesis elucidate that IL-16 is designated as a potential biomarker and treatment target according to urine proteomics and renal single-cell transcriptomics (26).

In this study, we first explored the differentially expressed genes (DEGs) between LN patients and healthy individuals based on the dataset GSE32591 acquired from the Gene Expression Omnibus (GEO) database. Next, we screened out the extracellular protein-differentially expressed genes (EP-DEGs) *via* the intersection between DEGs and extracellular proteins. A total of 164 DEGs were obtained, of which 38 DEGs were identified as EP-DEGs for further analysis. Gene Ontology (GO) and Kyoto Encyclopedia of Genes and Genomes (KEGG) analyses were subsequently conducted to identify the biological function and pathway enrichment. We further determined the key EP-DEGs as the potential biomarkers such as LUM, TGFBI, COL1A2, and POSTN *via* the establishment of the protein–protein interaction (PPI) network. Additionally, we investigated the expression level, immune characteristics, and diagnostic value of these candidate biomarkers. Finally, the correlations between these biomarkers and clinicopathological features such as sex, WHO class, and renal function were analyzed. The aim of this study was to explore key extracellular proteins that might serve as the potential biomarkers in the diagnosis and prognosis of LN patients, as well as the emerging therapeutic targets laying the foundation for improving the treatment effects.

## Materials and methods

### Data acquisition and process

**Figure 1:**
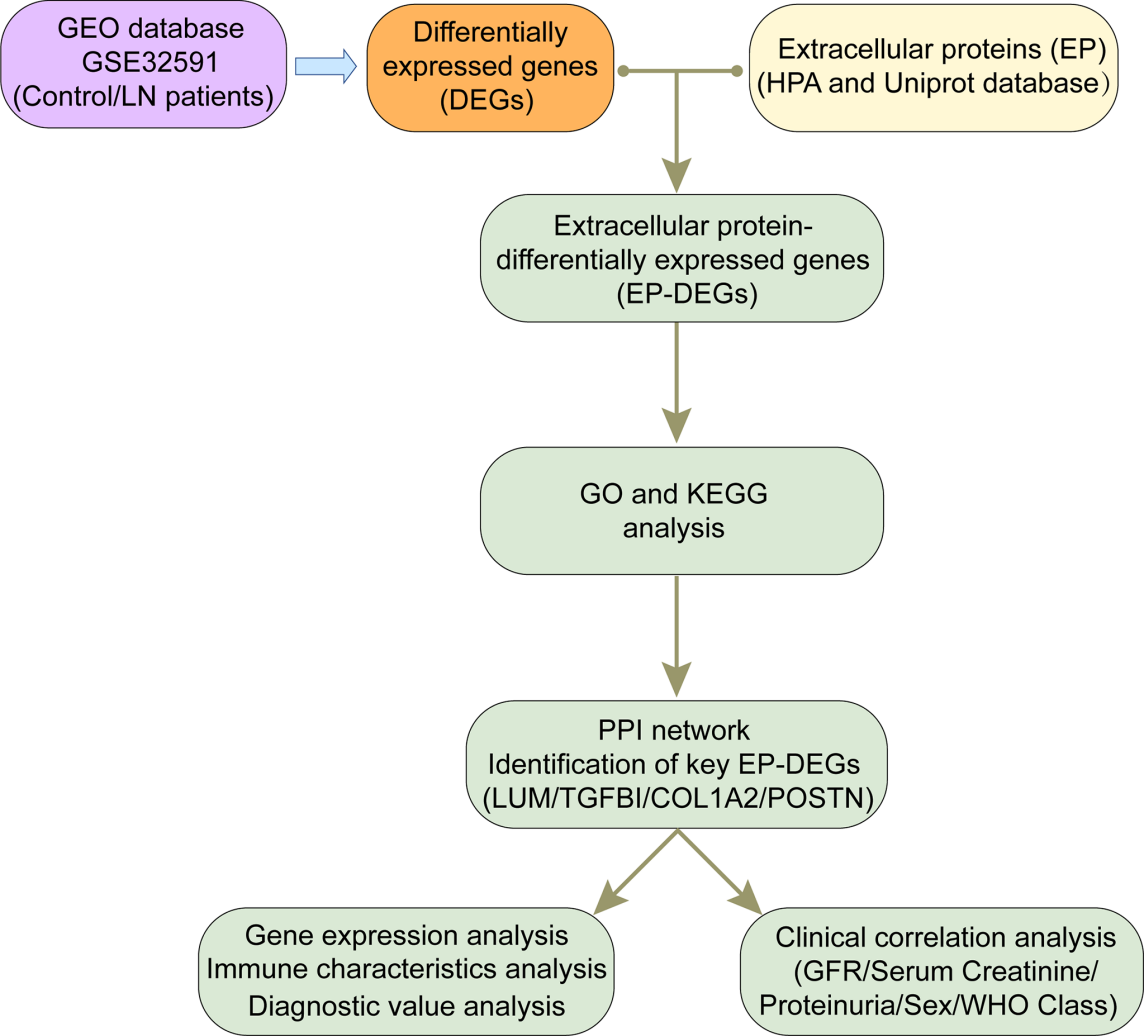
Flow diagram of the study.

The research strategy was shown as a flowchart in **Figure 1**. The dataset GSE32591 was obtained from the GEO database (http://www.ncbi.nlm.nih.gov/geo/). The platform for GSE32591 was GPL14663 (Affymetrix GeneChip Human Genome HG-U133A Custom CDF), which contained 64 LN kidney biopsy samples and 29 healthy control samples (27). Gene symbols were recognized through a normalized conversion of all probes according to the platform annotation information. Probes corresponding to more than one gene were eliminated, and the average value would be calculated for the case of genes corresponding to more than one probe. We used “limma” (28) and “sva” (29) packages to remove the batch effect. Principal component analysis (PCA) was conducted using the “PCA” package.

### Identification of DEGs

DEGs between the LN sample and control groups were identified utilizing the “Limma” package setting the criteria “adjusted *P* < 0.05 and Fold Change > 2,” which were exhibited *via* volcano plots and heatmaps.

### Screening of EP-DEGs

We downloaded two extracellular protein gene lists from the Human Protein Atlas (HPA) database (30) and the UniProt database (GO:0005576) (31), respectively (**Supplementary Data 1**). A Venn diagram (32) was utilized to separately perform the intersection between LN-DEGs and the two extracellular protein gene lists, then HPA-DEGs and UniProt-DEGs were identified. Here, we combined the results of two intersections (HPA-DEGs and UniProt-DEGs) to screen out EP-DEGs for further analysis.

### Function enrichment analysis

Gene enrichment analysis was performed using GO and KEGG by means of “ggplot2”, “GO plot”, and “Cluster Profiler” packages. We successively conducted GO and KEGG enrichment analyses of LN-DEGs and EP-DEGs. Furthermore, GO enrichment of EP-DEGs involved in the biological process (BP), cellular component (CC), and molecular function (MF) was exhibited using a circle graph.

### PPI network construction of EP-DEGs and identification of the key extracellular protein genes

A PPI network of EP-DEGs was created *via* the STRING website (https://string-db.org/) (33), which was visualized using Cytoscape (version 3.8.0). Then, the Molecular Complex Detection (MCODE) method was utilized to explore the functional gene clusters in the PPI network (34). Meanwhile, the top 10 node genes were respectively acquired according to the score calculated by “Maximal Clique Centrality (MCC)” and “Degree” methods using the CytoHubba plug-in *via* Cytoscape. Finally, the intersection of these genes obtained from the three methods (MCODE, MCC, and Degree) was taken to identify the key EP-DEGs, which were displayed *via* a Venn diagram.

### Gene expression, immune characteristics, and diagnostic value analysis

We first investigated the expression levels of the key EP-DEGs between LN samples and controls. Furthermore, the correlations between the expression of key EP-DEGs and immunoregulatory genes such as chemokines, chemokine receptors, and MHC genes were analyzed by Spearman’s correlation analysis. In addition, the receiver operating characteristic (ROC) curve was applied to assess the diagnostic value of the key EP-DEGs in LN patients, which was conducted *via* the “pROC” package. Then, we calculated the value of the area under the curve (AUC). The higher the AUC value is, the better the diagnostic value is. Generally, an AUC value of 0.5–0.7 reveals a low effect, an AUC value of 0.7–0.9 reveals a middle effect, and an AUC value above 0.9 reveals a high effect.

### Clinical correlation analysis

The Nephroseq V5 tool (http://v5.nephroseq.org/) was utilized to investigate the relevance between the expression of the key EP-DEGs and the clinicopathological features such as sex, WHO class, and renal function indexes in the patients with LN. Then, visualization was performed utilizing the “ggplot2” package.

### Statistics analysis

Student’s t-test or Wilcoxon rank-sum test was applied to assess differences between the two groups. Correlations between gene expression and clinical data were conducted by Spearman’s correlation analysis. All R packages mentioned above were operated under R software version v4.0.3, and statistical significance was acknowledged in case of *P* < 0.05.

## Results

### Identification of DEGs and biological function

**Figure 2:**
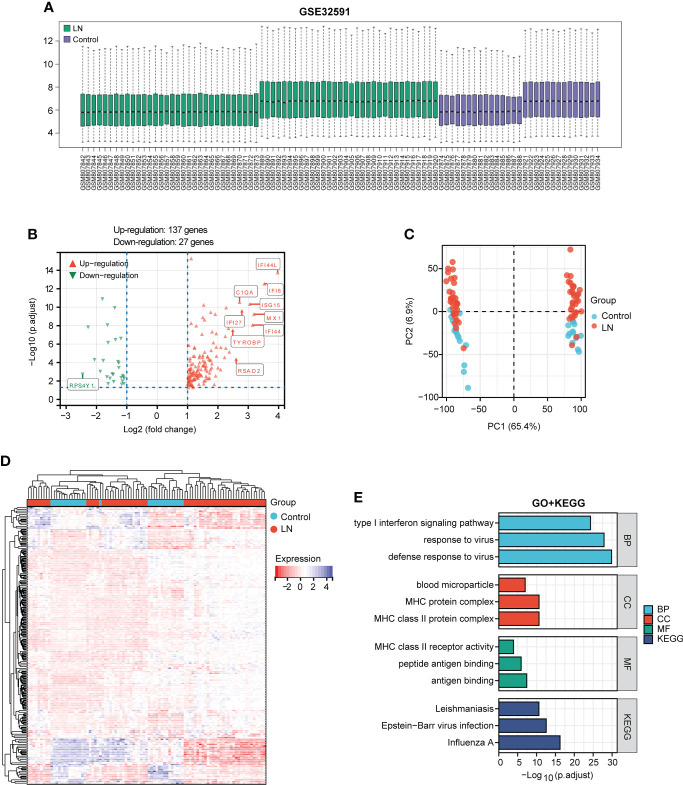
Identification of DEGs between the LN sample and control groups in the GSE32591 dataset and function enrichment analysis. **(A)** Box plot displaying gene probe expression levels. **(B)** Volcano plot of DEGs between LN patients and controls. The top 10 differentially expressed genes are marked on the plot. **(C)** PCA of the samples. **(D)** Expression heatmap of all screened DEGs. **(E)** GO and KEGG enrichment analyses of DEGs.

After standardizing the microarray data of GSE32591 (**Figure 2A**), we identified 164 DEGs in GSE32591, which contained 137 upregulated and 27 downregulated differential genes (**Figure 2B**; **Supplementary Data 2**). The significant differences between the LN and control groups were confirmed by PCA (**Figure 2C**). Subsequently, we performed hierarchical clustering analysis for displaying DEGs by heatmap (**Figure 2D**). Additionally, we further performed KEGG and GO enrichment analyses, which indicated that DEGs primarily participated in defense response to virus, MHC protein complex, antigen binding, and influenza A (**Figure 2E**).

### Screening of EP-DEGs

**Figure 3:**
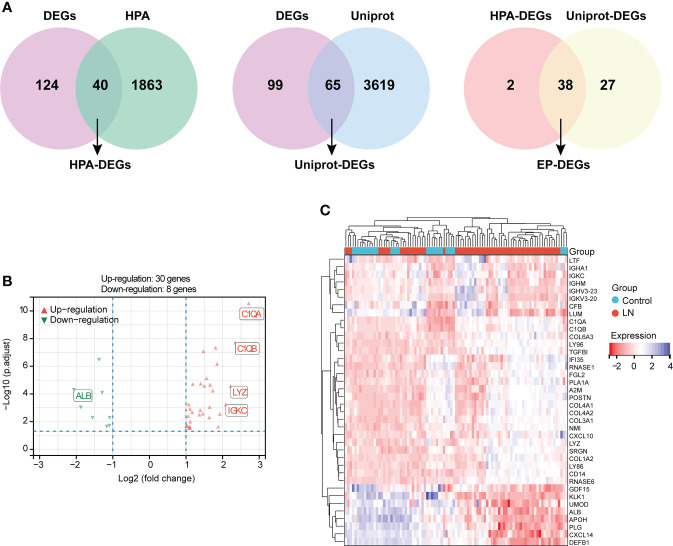
Identification of DEGs encoding extracellular proteins (EP-DEGs). **(A)** Venn diagram of 40 HPA-DEGs obtained based on the intersection between HPA database-extracellular proteins and LN-DEGs. Meanwhile, a Venn diagram of 65 UniProt-DEGs obtained based on the intersection between UniProt database-extracellular proteins and LN-DEGs. Finally, 38 EP-DEGs were screened out based on the intersection between HPA-DEGs and UniProt-DEGs. **(B)** Volcano plot of EP-DEGs between LN patients and controls. **(C)** Expression heatmap of screened EP-DEGs.

A total of 40 HAP-DEGs and 65 UniProt-DEGs were screened out by means of a Venn diagram, and 38 EP-DEGs were determined after the intersection between HAP-DEGs and UniProt-DEGs (**Figure 3A**). The distribution of EP-DEGs was depicted by the volcano plot shown in **Figure 3B**, containing 30 upregulated and eight downregulated genes (**Supplementary Data 3** **)**. The top 20 EP-DEGs ranked by “adj.P.Value” are displayed in **Table 1**. The expression heatmap of EP-DEGs is exhibited in **Figure 3C**.

**Table 1:** The top 20 EP-DEGs between the LN sample and control groups in GSE32591.

Gene symbol	logFC	AveExpr	adj.P.Value
C1QA	2.712928	7.708666	2.85E-11
C1QB	2.346039	7.491719	1.92E-08
LY96	1.806014	5.82231	4.58E-08
TGFBI	1.474979	7.952755	8.3E-08
KLK1	-1.37425	7.726793	3.39E-07
COL6A3	1.849268	6.625157	6.77E-07
NMI	1.560511	8.064883	7.24E-06
FGL2	1.401285	9.054541	1.82E-05
CFB	1.184815	7.391285	2E-05
RNASE6	1.656417	6.609036	2.22E-05
CD14	1.497218	8.287886	2.68E-05
LYZ	2.21924	7.454621	2.75E-05
ALB	-2.06514	8.569382	4.99E-05
COL1A2	1.73149	7.048733	5.77E-05
GDF15	-1.29125	7.579357	8.17E-05
IGKC	2.087748	10.6152	0.000572
LY86	1.074905	7.441597	0.00058
COL3A1	1.646891	8.054652	0.000887
UMOD	-1.87458	10.65982	0.000945
COL4A2	1.107447	8.005439	0.001234

AveExpr represents the mean expression level of the gene in all samples. The rank of the top 20 EP-DEGs is based on adj.P.Value.

### Function enrichment analysis of EP-DEGs

**Figure 4:**
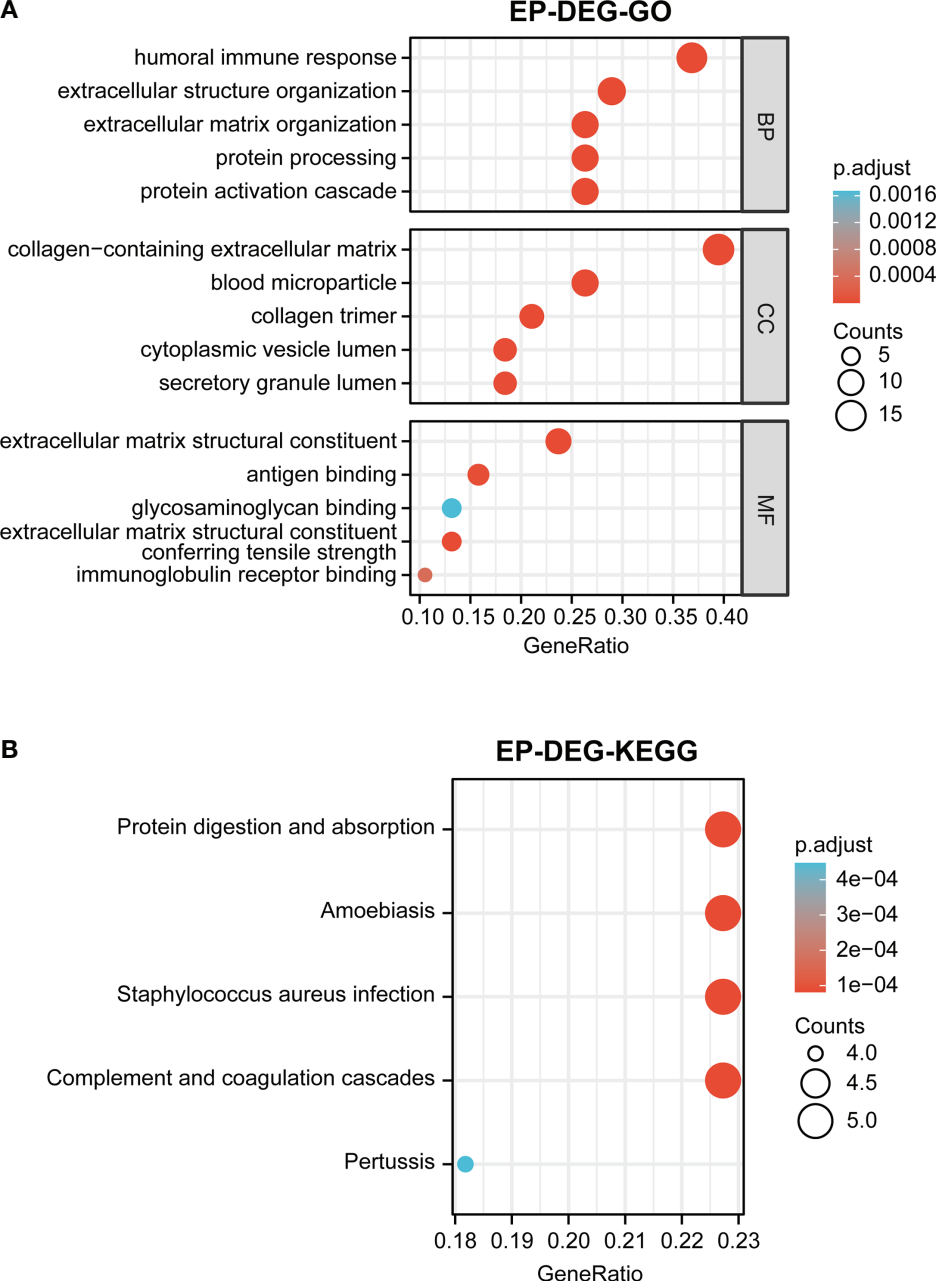
Function enrichment analysis of EP-DEGs. **(A)** GO enrichment analysis of EP-DEGs in BP, CC, and MF processes (BP, biological process; CC, cellular component; MF, molecular function). **(B)** KEGG enrichment analysis of EP-DEGs. The size of the circles indicates the number of genes. The larger the circle, the greater the number of genes.

**Figure 5:**
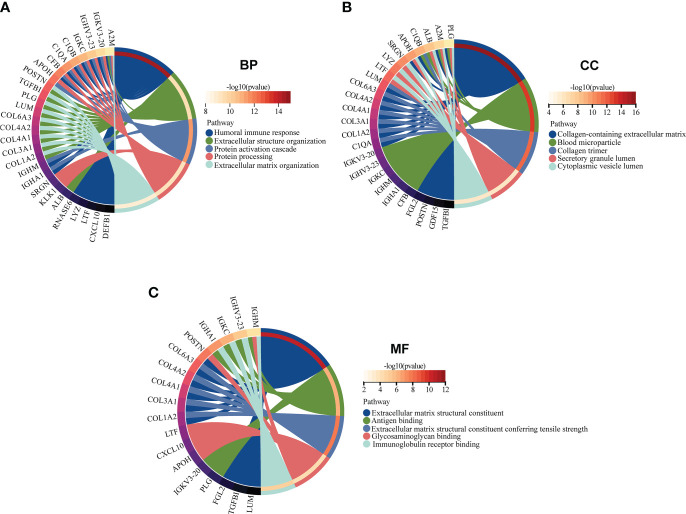
GO enrichment analysis of EP-DEGs in BP, CC, and MF (BP, biological process; CC, cellular component; MF, molecular function). **(A–C)** The circle graph showing the top5 pathway of BP, CC, and MF, respectively. Different color represents different pathway, and the differentially expressed genes in the pathway are displayed.

GO and KEGG enrichment analyses of 38 EP-DEGs were performed respectively. The results of GO enrichment analysis indicated that EP-DEGs were chiefly enriched in humoral immune response, extracellular structure organization, collagen-containing extracellular matrix, and extracellular matrix structural constituent (**Figure 4A**). Simultaneously, KEGG analysis confirmed that EP-DEGs were mostly enriched in protein digestion and absorption, amoebiasis, staphylococcus aureus infection, and complement and coagulation cascades (**Figure 4B**). Furthermore, a circle graph was applied for exhibiting the top five pathways of BP, CC, and MF, respectively (**Figures 5A–C**).

### Construction of the PPI network and identification of hub genes

**Figure 6:**
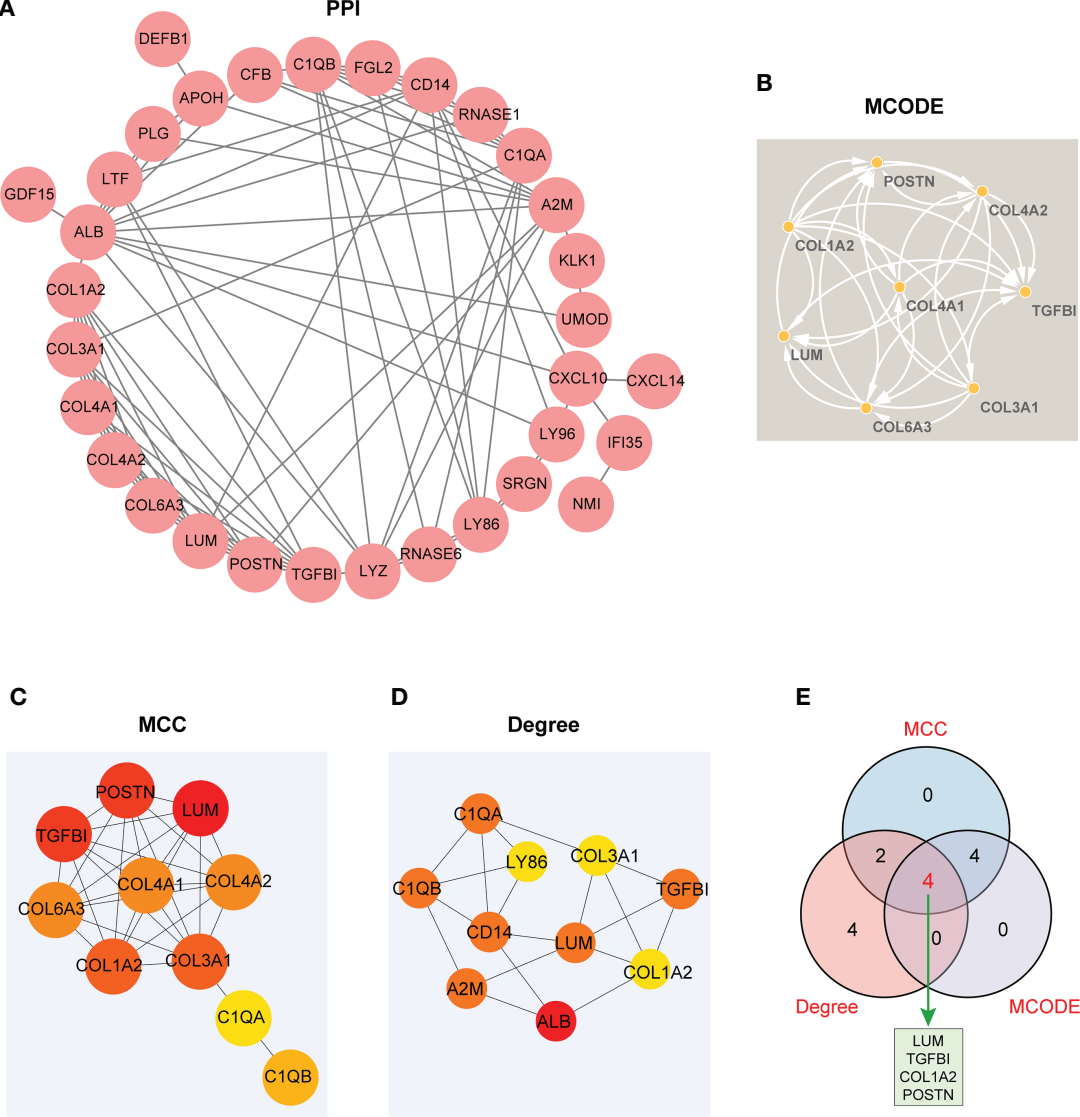
The visualized PPI network of EP-DEGs for determining the key EP-DEGs. **(A)** Construction of the PPI network of 38 EP-DEGs *via* the STRING database. **(B)** A total of eight candidate genes were identified from EP-DEGs according to the score sorted by the MCODE method using Cytoscape. **(C)** Top 10 genes were obtained according to the score calculated by the MCC method. **(D)** Top 10 genes were selected according to the score calculated by the Degree method. **(E)** Venn diagram displaying four key genes such as LUM, TGFBI, COL1A2, and POSTN acquired by the intersection of the three methods (MCC, Degree, and MCODE). The other genes obtained from the intersection between MCC and Degree methods were C1QB and C1QA. The other genes obtained from the intersection between MCC and MCODE methods were COL3A1, COL6A3, COL4A2, and COL4A1.

A PPI network involving 38 EP-DEGs was constructed utilizing STRING and visualized by Cytoscape (**Figure 6A**). Subsequently, we explored functional modules by means of the MCODE plug-in *via* Cytoscape, and a total of eight candidate genes were screened out from EP-DEGs (**Figure 6B**). Meanwhile, top 10 hub genes were obtained by two topological methods including MCC and Degree, utilizing the CytoHubba plug-in *via* Cytoscape (**Figures 6C, D** **)**. The detailed gene information of top 10 EP-DEGs is exhibited in **Table 2**. Finally, four key genes such as LUM, TGFBI, COL1A2, and POSTN were determined by the intersection of the three methods (**Figure 6E**).

**Table 2:** The top 10 EP-DEGs ranked by MCC and Degree method using the CytoHubba plug-in *via* Cytoscape.

Rank	MCC	Degree
1	LUM	ALB
2	TGFBI	A2M
3	POSTN	LUM
4	COL1A2	TGFBI
5	COL3A1	C1QB
6	COL6A3	CD14
7	COL4A2	C1QA
8	COL4A1	LYZ
9	C1QB	POSTN
10	C1QA	COL1A2

### Gene expression analysis

**Figure 7:**
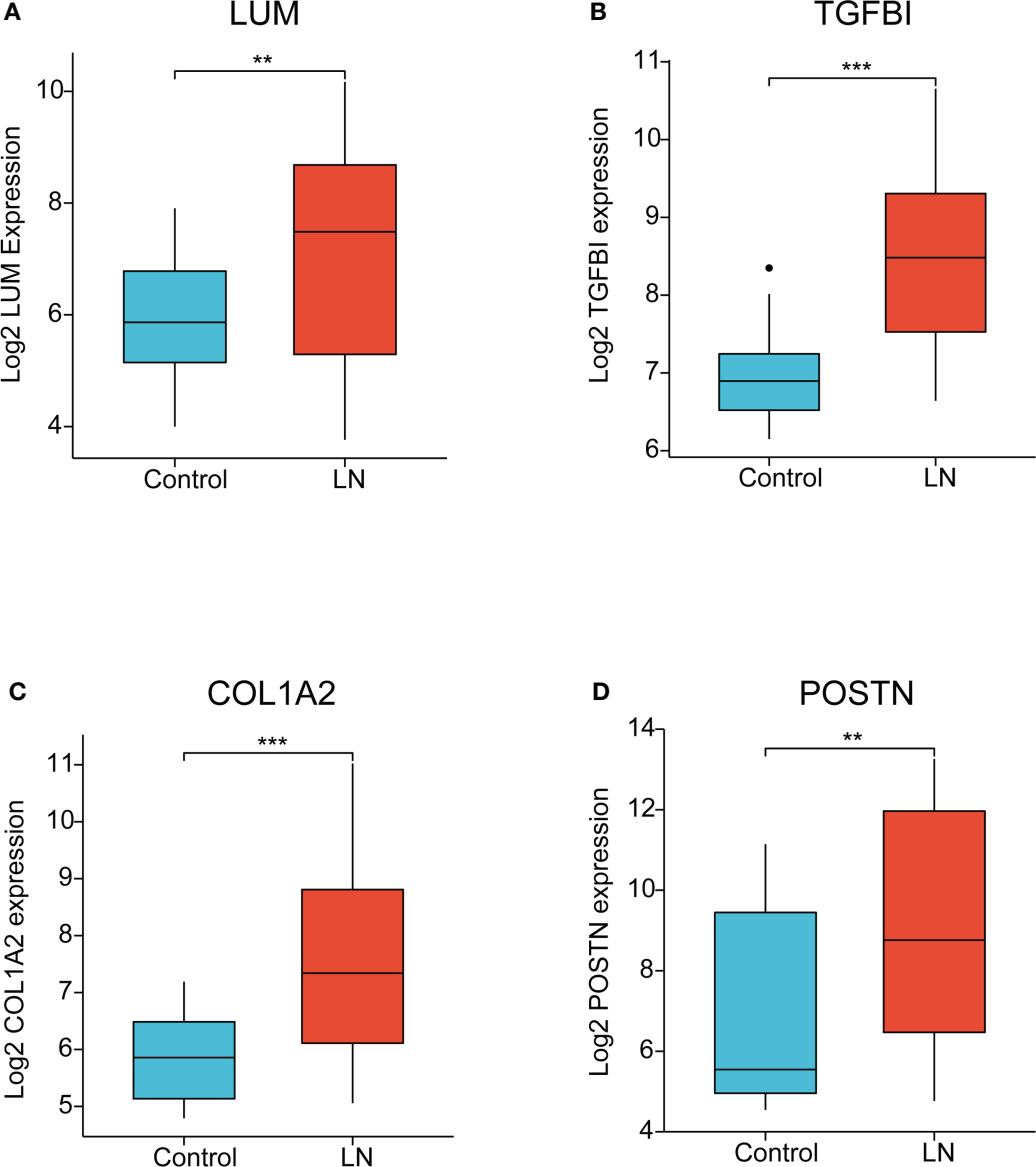
The expression of key EP-DEGs identified as the potential biomarkers. **(A-D)** The expression level of LUM, TGFBI, COL1A2, and POSTN between LN and control groups, respectively. (**P < 0.01 and ***P < 0.001).

The four EP-DEGs (LUM, TGFBI, COL1A2, POSTN) were defined as the potential biomarkers. We first evaluated the expression level of the four biomarkers between LN and healthy controls. As shown in **Figures 7A–D**, the four genes were all dramatically upregulated in LN samples, compared to the controls.

### Immune characteristics analysis of the potential biomarkers

**Figure 8:**
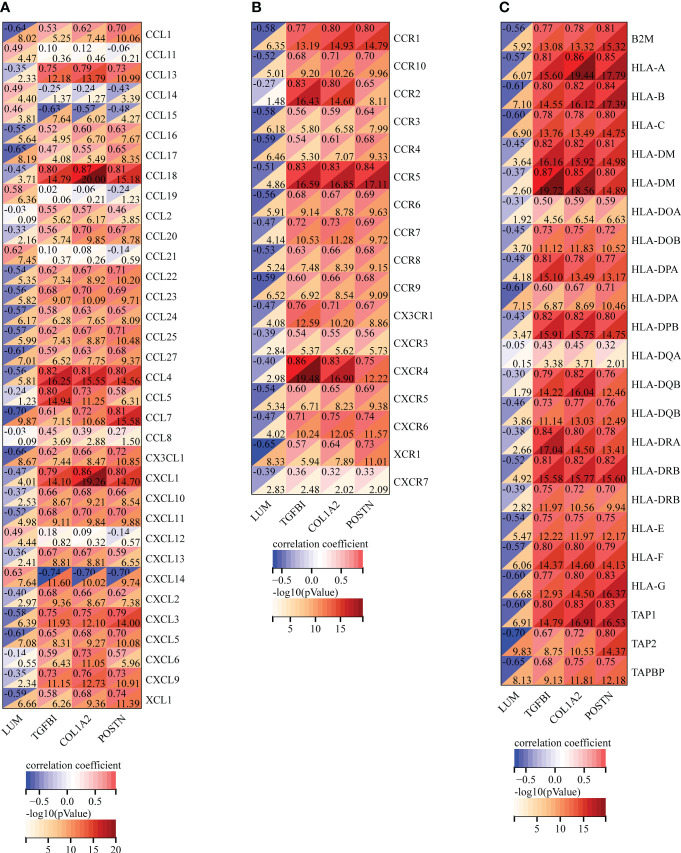
Correlation analysis of the expression of LUM, TGFBI, COL1A2, and POSTN with immunoregulatory genes, including **(A)** chemokines, **(B)** chemokine receptors, and **(C)** MHC genes.

To further ascertain the immune characteristics of these potential biomarkers, we performed the correlation analyses between the key EP-DEG expression and immunoregulatory genes, including chemokines (**Figure 8A** **)**, chemokine receptors (**Figure 8B**), and MHC genes (**Figure 8C**) in LN samples. Notably, we uncovered that LUM expression was negatively associated with most immune regulation genes in LN. However, the expression levels of TGFBI, COL1A2, and POSTN were all positively associated with most of these immune regulation genes. Taken together, the four candidate biomarkers exhibited potential immune properties, suggesting that these EP-DEGs might exert a crucial role in immune regulation in LN.

### Diagnostic value analysis of the potential biomarkers in LN

**Figure 9:**
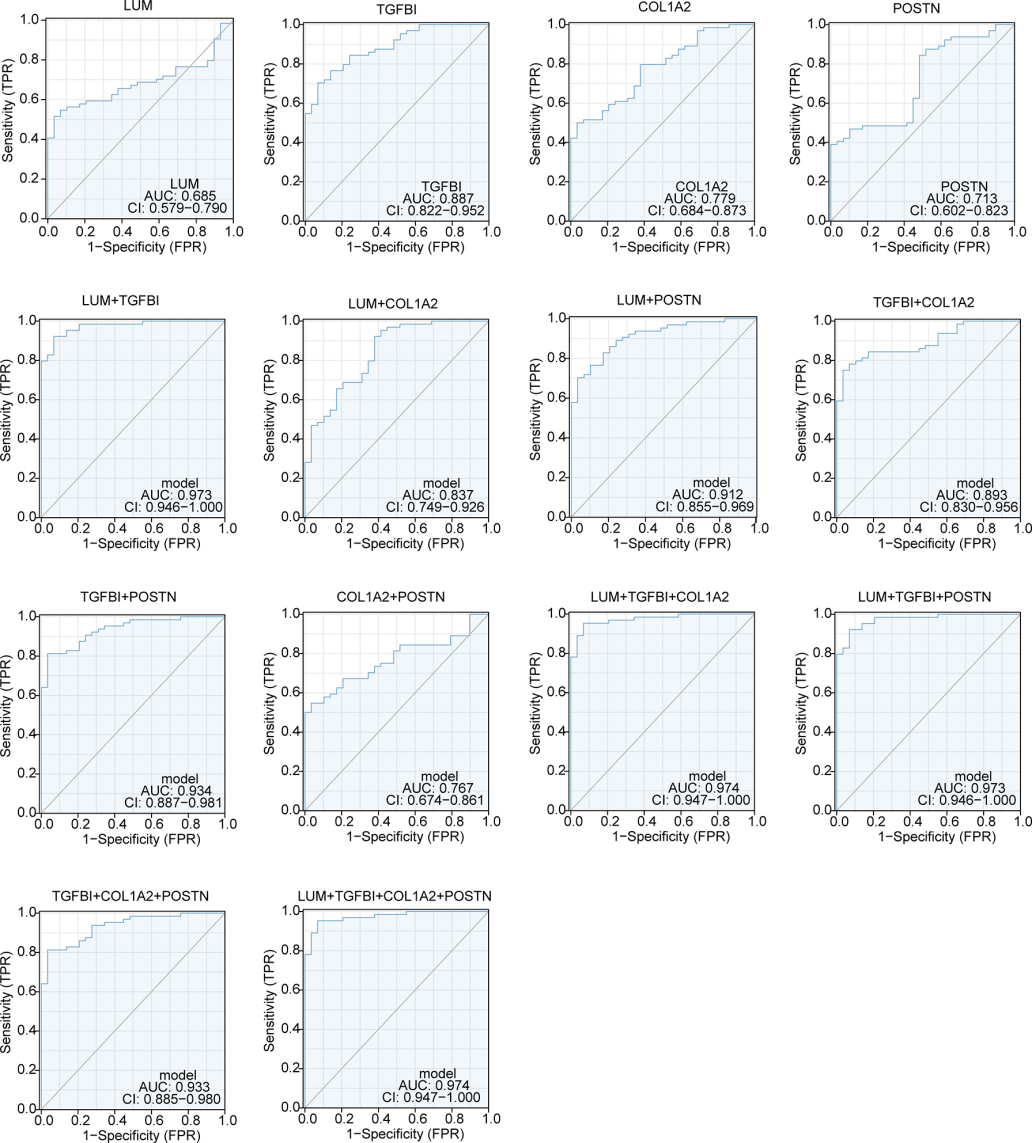
The diagnostic value of LUM, TGFBI, COL1A2, and POSTN in LN patients.

AUC, an indicator of inherent validity of diagnostic tests, is characterized with sensitivity and specificity (35). The AUC value of single genes had a moderate accuracy in the diagnosis of LN, such as LUM (AUC = 0.685), TGFBI (AUC = 0.887), COL1A2 (AUC = 0.779), and POSTN (AUC = 0.713). However, the AUC values of any combination of the two genes were significantly increased within a certain range (0.767–0.973). The AUC value could exceed 0.9 by any combination of three genes. Notably, the AUC value of LUM-TGFBI provided a significant boost in prediction performance (AUC = 0.973). In addition, the AUC value of the LUM–TGFBI–COL1A2 combination already reached 0.974, which was the same as the value of the four gene combinations (LUM–TGFBI–COL1A2–POSTN) (**Figure 9**). In summary, the four EP-DEGs had high AUC values for the diagnosis of LN patients, especially the combination of LUM-TGFBI, which indicated that they could serve as potential diagnostic biomarkers for LN patients.

### Correlation analysis of the key extracellular proteins with the clinicopathological features in LN

**Figure 10:**
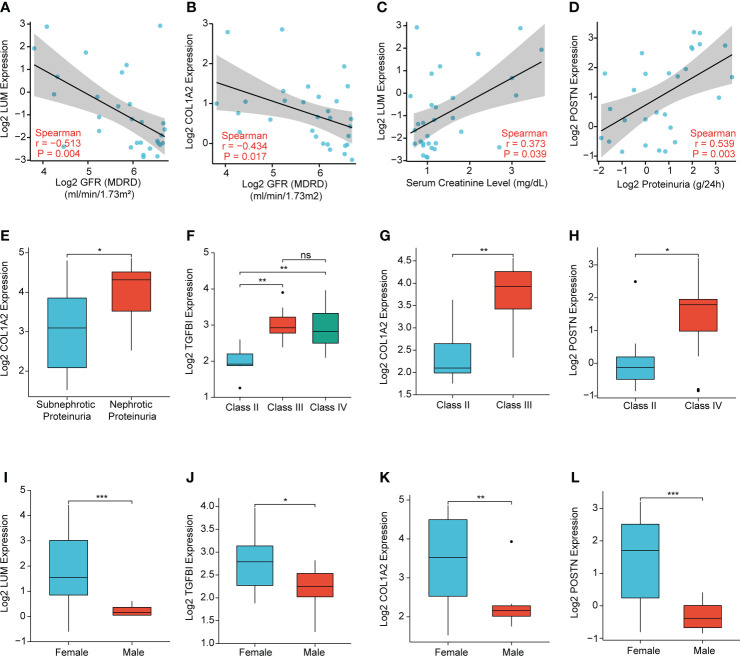
Correlation analysis of the expression levels of LUM, TGFBI, COL1A2, and POSTN with the clinicopathological features, including **(A, B)** glomerular filtration rate (GFR), **(C)** serum creatinine level, **(D, E)** proteinuria, **(F–H)** WHO class, and **(I-L)** sex. (*P < 0.05, **P < 0.01, ***P < 0.001 and ns, no significance).

To further illuminate the clinical significance of the key extracellular proteins in LN, we conducted the correlation analyses of LUM, TGFBI, COL1A2, and POSTN expression with several clinicopathological features based on the Nephroseq database. The results demonstrated that LUM/COL1A2 expression was negatively correlated with glomerular filtration rate (GFR) (**Figure 10A, B** **)**. Meanwhile, there was a significantly positive correlation between LUM expression and serum creatinine level (**Figure 10C**). Additionally, a high POSTN/COL1A2 expression was predictive of increased proteinuria (**Figures 10D, E** **)**. In addition, TGFBI/COL1A2/POSTN expression was upregulated in the higher WHO class of LN patients (**Figures 10F-H**), which indicated that these genes might predict a worse kidney damage in patients with LN. Finally, we detected a connection of high LUM/TGFBI/COL1A2/POSTN expression to female LN patients (**Figures 10I-L**).

## Discussion

LN, a severe complication of SLE, is shown to be the leading cause of morbidity and mortality in SLE owing to the progression of ESRD (36). The histopathologic examination of kidney biopsies in LN has an indispensable role in diagnosis and assessment of therapeutic outcomes. However, there are also limitations to kidney biopsies. The problems of kidney biopsy involving bleeding risk, infection, and other puncture complications are well known. In addition, histology does not identify the potential change of specific active biological pathways. Histological class transformations are common when performing the repeat renal biopsy (37). Sometimes, biopsy specimen is unobtainable, and the sample may not be representative of the entire kidney. Since kidney biopsy is a relative hysteretic indicator, there is a pressing need for a less invasive and easy-to-access biomarker with high diagnostic value.

Leveraging differentially expressed extracellular proteins in LN samples and healthy controls, our study identified the key EP-DEGs as the potential biomarkers for diagnosis and clinical outcomes of LN patients. We first found the following: (1) there were 164 DEGs between LN samples and healthy controls based on the GSE32591 dataset, including 137 upregulated and 27 downregulated differential genes; (2) 38 EP-DEGs were screened out through the intersection between DEGs and extracellular protein gene lists. GO enrichment analysis determined that these EP-DEGs might participate in immune response and constitute the extracellular matrix in the onset and development of LN. In addition, the circle graph also displayed EP-DEGs enriched with multiple biological processes, including humoral immune response, extracellular structure organization, protein activation cascade, protein processing, and extracellular matrix organization, which further specified the potential biological function of these EP-DEGs.

After the intersection of three gene sets obtained from different methods (MCODE, MCC, and Degree) based on the PPI network, four key EP-DEGs (LUM, TGFBI, COL1A2, and POSTN) were eventually identified as candidate biomarkers. Until now, however, the potential biological function of the four candidate biomarkers in LN has never been fully understood, but they have been reported in several other diseases. As a member of small leucine-rich proteoglycans, lumican (LUM) has been reported to participate in a variety of biological processes, including tissue homeostasis, cell proliferation, differentiation, and extracellular matrix (ECM) remodeling (38, 39). Research has indicated that polymorphisms in LUM are linked with the development and clinical manifestations of SLE (40). A study has confirmed that TGFBI serving as a ubiquitination substrate is reduced in clear cell renal cell carcinoma (ccRCC) (41). As a member of the collagen family, COL1A2 is significantly upregulated in metastatic ccRCC (42). Moreover, periostin (POSTN) has been identified as one of key genes and is significantly upregulated in IgA nephropathy (IgAN) with enhancing mesangial cell proliferation (43).

Recent research has indicated that the expression of COL1A2 and the pro-inflammatory cytokines is significantly increased in mesangial cells under inflammation conditions in LN, which participates in LN-associated fibrosis (44). Furthermore, periostin plays a crucial role in the platelet-derived growth factor (PDGF)-induced proliferation of mesangial cells and the accumulation of the extracellular matrix in lupus nephritis (45). Moreover, periostin has been identified as a novel biomarker, which is linked with the chronicity index and renal function of the patients with LN (46). Thus, COL1A2 and POSTN perform a vital role in the pathogenesis of LN and also serve as promising therapeutic targets.

Using identification of the extracellular protein approach, we discovered the four unrecognized biomarkers (LUM, TGFBI, COL1A2, and POSTN). It is noteworthy that these biomarkers were all overexpressed in LN samples compared to the control group. Meanwhile, the immune characteristics of these potential biomarkers were further analyzed. We uncovered that there was a good correlation between the four EP-DEGs and immune regulation genes, including chemokines, chemokine receptors, and MHC genes in LN, suggesting that the four EP-DEGs possessed strong immune characteristics. This was consistent with chemokine/chemokine receptor expression observed in LN patients contributing to macrophage infiltration and influencing the inflammatory process (47, 48). Furthermore, the diagnostic value of these biomarkers was also investigated. We were surprised to find that the AUC value of the LUM–TGFBI combination reached almost 1 (AUC = 0.973), which demonstrated high accuracy in distinguishing LN from controls. The ideal biomarkers in LN could accurately detect the clinical changes of nephritis activity and longitudinally monitor posttreatment effects. We further conducted the correlation analysis of the four biomarkers with several clinicopathological features to elucidate the clinical significance of these candidates in LN patients. Notably, we found a meaningful correlation of these biomarkers with sex, WHO class, and renal function such as GFR, serum creatinine level, and proteinuria. Overall, these differentially expressed extracellular proteins were first identified in LN diagnosis and pathogenesis and thus considered potential biomarkers and therapeutic targets.

We acknowledged that our study had some limitations. First, despite that we collected as many samples as possible in this research, it was also limited by the small sample size (64 LN, 29 controls). Therefore, future work with a larger sample size is required to validate this trend. Second, four key EP-DEGs (LUM, TGFBI, COL1A2, and POSTN) were eventually identified as candidate biomarkers based on the bioinformatics analysis, which were also needed to be further confirmed experimentally. Meanwhile, additional research is further performed to confirm the diagnostic value and clinical significance of these candidates in blood and urine specimens. However, our study revealed that the four key EP-DEGs had high diagnostic value in LN patients, and their potential biological function was further explored *via* the Nephroseq clinical database. Thus, it is reasonable to think that the four key EP-DEGs play a potential role in LN diagnosis and pathogenesis and are expected to be therapeutic targets. Further research with sufficient clinical samples will be carried out to identify the clinical evidence of these biomarkers in LN patients.

In summary, this study linked extracellular proteins with lupus nephritis-related DEGs, identifying the potential roles of the four key EP-DEGs in LN diagnosis and pathogenesis, and promising EP-DEGs as therapeutic targets. Further research is needed to complement and determine the clinical applicability of these candidates in LN.

## Data availability statement

Publicly available datasets were analyzed in this study. This data can be found here: https://www.ncbi.nlm.nih.gov/geo/query/acc.cgi?acc=GSE32591.

## Author contributions

XZ and NW designed the project. XZ prepared the manuscript, and NW revised the manuscript. All authors contributed to data analysis and integration, visualization, and figure generation and approved the submitted version of the article.

## Funding

This work was sponsored by Tianjin Health Research Project (Grant No. TJWJ2022QN076).

## Conflict of interest

The authors declare that the research was conducted in the absence of any commercial or financial relationships that could be construed as a potential conflict of interest.

## Publisher’s note

All claims expressed in this article are solely those of the authors and do not necessarily represent those of their affiliated organizations, or those of the publisher, the editors and the reviewers. Any product that may be evaluated in this article, or claim that may be made by its manufacturer, is not guaranteed or endorsed by the publisher.

## Abbreviations

AUC, area under the curve; BP, biological process; CC, cellular component; ccRCC, clear cell renal cell carcinoma; DEGs, differentially expressed genes; ECM, extracellular matrix; EP-DEGs, extracellular protein-differentially expressed genes; ESRD, end-stage renal disease; GEO, gene expression omnibus; GFR, glomerular filtration rate; GO, Gene Ontology; HPA, human protein atlas; IgAN, IgA nephropathy; KEGG, Kyoto Encyclopedia of Genes and Genomes; LN, lupus nephritis; MCC, maximal clique centrality; MCODE, molecular complex detection; MF, molecular function; PCA, principal components analysis; PPI, protein–protein interaction; ROC, receiver operating characteristic; SLE, systemic lupus erythematosus.

## References

[1] Anders HJ Saxena R Zhao MH Parodis I Salmon JE Mohan C. Lupus nephritis. Nat Rev Dis Primers (2020) 6(1):7. doi: 10.1038/s41572-019-0141-9 31974366

[2] Almaani S Meara A Rovin BH. Update on lupus nephritis. Clin J Am Soc Nephrol (2017) 12(5):825–35. doi: 10.2215/CJN.05780616 PMC5477208 27821390

[3] Brunner HI Gladman DD Ibañez D Urowitz MD Silverman ED. Difference in disease features between childhood-onset and adult-onset systemic lupus erythematosus. Arthritis Rheumatol (2008) 58(2):556–62. doi: 10.1002/art.23204 18240232

[4] Hoover PJ Costenbader KH. Insights into the epidemiology and management of lupus nephritis from the US rheumatologist's perspective. Kidney Int (2016) 90(3):487–92. doi: 10.1016/j.kint.2016.03.042 PMC5679458 27344205

[5] Houssiau FA. Biologic therapy in lupus nephritis. Nephron Clin Pract (2014) 128(3-4):255–60. doi: 10.1159/000368587 25401689

[6] Maria NI Davidson A. Protecting the kidney in systemic lupus erythematosus: From diagnosis to therapy. Nat Rev Rheumatol (2020) 16(5):255–67. doi: 10.1038/s41584-020-0401-9 32203285

[7] Davidson A. What is damaging the kidney in lupus nephritis. Nat Rev Rheumatol (2016) 12(3):143–53. doi: 10.1038/nrrheum.2015.159 PMC4820834 26581344

[8] Thacker SG Berthier CC Mattinzoli D Rastaldi MP Kretzler M Kaplan MJ. The detrimental effects of IFN-α on vasculogenesis in lupus are mediated by repression of IL-1 pathways: Potential role in atherogenesis and renal vascular rarefaction. J Immunol (2010) 185(7):4457–69. doi: 10.4049/jimmunol.1001782 PMC2978924 20805419

[9] Kahlenberg JM Kaplan MJ. The inflammasome and lupus: another innate immune mechanism contributing to disease pathogenesis. Curr Opin Rheumatol (2014) 26(5):475–81. doi: 10.1097/BOR.0000000000000088 PMC4153426 24992143

[10] Thanei S Vanhecke D Trendelenburg M. Anti-C1q autoantibodies from systemic lupus erythematosus patients activate the complement system *via* both the classical and lectin pathways. Clin Immunol (2015) 160(2):180–7. doi: 10.1016/j.clim.2015.06.014 26148903

[11] Burbano C Gómez-Puerta JA Muñoz-Vahos C Vanegas-García A Rojas M Vásquez G. HMGB1+ microparticles present in urine are hallmarks of nephritis in patients with systemic lupus erythematosus. Eur J Immunol (2019) 49(2):323–35. doi: 10.1002/eji.201847747 30537116

[12] Kitagawa A Tsuboi N Yokoe Y Katsuno T Ikeuchi H Kajiyama H. Urinary levels of the leukocyte surface molecule CD11b associate with glomerular inflammation in lupus nephritis. Kidney Int (2019) 95(3):680–92. doi: 10.1016/j.kint.2018.10.025 30712924

[13] Stanley S Mok CC Vanarsa K Habazi D Li J Pedroza C. Identification of low-abundance urinary biomarkers in lupus nephritis using electrochemiluminescence immunoassays. Arthritis Rheumatol (2019) 71(5):744–55. doi: 10.1002/art.40813 30618193

[14] Mok CC Soliman S Ho LY Mohamed FA Mohamed FI Mohan C. Urinary angiostatin, CXCL4 and VCAM-1 as biomarkers of lupus nephritis. Arthritis Res Ther (2018) 20(1):6. doi: 10.1186/s13075-017-1498-3 29325582 PMC5765646

[15] Ding Y Nie LM Pang Y Wu WJ Tan Y Yu F. Composite urinary biomarkers to predict pathological tubulointerstitial lesions in lupus nephritis. Lupus (2018) 27(11):1778–89. doi: 10.1177/0961203318788167 30020021

[16] Zhang ZY Ravassa S Pejchinovski M Yang WY Zürbig P López B. A urinary fragment of mucin-1 subunit α is a novel biomarker associated with renal dysfunction in the general population. Kidney Int Rep (2017) 2(5):811–20. doi: 10.1016/j.ekir.2017.03.012 PMC5589115 28920100

[17] Hauschke M Roušarová E Flídr P Čapek J Libra A Roušar T. Neutrophil gelatinase-associated lipocalin production negatively correlates with HK-2 cell impairment: Evaluation of NGAL as a marker of toxicity in HK-2 cells. Toxicol In Vitro (2017) 39:52–7. doi: 10.1016/j.tiv.2016.11.012 27888128

[18] Li H Zeng RL Liao YF Fu MF Zhang H Wang LF. Association of plasma connective tissue growth factor levels with hyperthyroid heart disease. Curr Med Sci (2021) 41(2):348–55. doi: 10.1007/s11596-021-2354-x 33877553

[19] Kikuchi N Satoh K Kurosawa R Yaoita N Elias-Al-Mamun M Siddique MAH. Selenoprotein p promotes the development of pulmonary arterial hypertension: Possible novel therapeutic target. Circulation (2018) 138(6):600–23. doi: 10.1161/CIRCULATIONAHA.117.033113 29636330

[20] Oh IJ Kim HE Song SY Na KJ Kim KS Kim YC. Diagnostic value of serum glutathione peroxidase 3 levels in patients with lung cancer. Thorac Cancer (2014) 5(5):425–30. doi: 10.1111/1759-7714.12113 PMC4704365 26767034

[21] Qian X Li C Pang B Xue M Wang J Zhou J. Spondin-2 (SPON2), a more prostate-cancer-specific diagnostic biomarker. PLoS One (2012) 7(5):e37225. doi: 10.1371/journal.pone.0037225 22615945 PMC3352876

[22] Kosnopfel C Sinnberg T Sauer B Niessner H Muenchow A Fehrenbacher B. Tumour progression stage-dependent secretion of YB-1 stimulates melanoma cell migration and invasion. Cancers (Basel) (2020) 12(8):2328. doi: 10.3390/cancers12082328 PMC7464723 32824741

[23] Chen J Xi B Zhao Y Yu Y Zhang J Wang C. High-mobility group protein B1 (HMGB1) is a novel biomarker for human ovarian cancer. Gynecol Oncol (2012) 126(1):109–17. doi: 10.1016/j.ygyno.2012.03.051 22484401

[24] Harris HE Andersson U Pisetsky DS. HMGB1: A multifunctional alarmin driving autoimmune and inflammatory disease. Nat Rev Rheumatol (2012) 8(4):195–202. doi: 10.1038/nrrheum.2011.222 22293756

[25] Lindau D Mussard J Rabsteyn A Ribon M Kötter I Igney A. TLR9 independent interferon α production by neutrophils on NETosis in response to circulating chromatin, a key lupus autoantigen. Ann Rheum Dis (2014) 73(12):2199–207. doi: 10.1136/annrheumdis-2012-203041 24013727

[26] Fava A Rao DA Mohan C Zhang T Rosenberg A Fenaroli P. Urine proteomics and renal single-cell transcriptomics implicate interleukin-16 in lupus nephritis. Arthritis Rheumatol (2022) 74(5):829–39. doi: 10.1002/art.42023 PMC9050800 34783463

[27] Berthier CC Bethunaickan R Gonzalez-Rivera T Nair V Ramanujam M Zhang W. Cross-species transcriptional network analysis defines shared inflammatory responses in murine and human lupus nephritis. J Immunol (2012) 189(2):988–1001. doi: 10.4049/jimmunol.1103031 22723521 PMC3392438

[28] Ritchie ME Phipson B Wu D Hu Y Law CW Shi W. Limma powers differential expression analyses for RNA-sequencing and microarray studies. Nucleic Acids Res (2015) 43(7):e47. doi: 10.1093/nar/gkv007 25605792 PMC4402510

[29] Parker HS Leek JT Favorov AV Considine M Xia X Chavan S. Preserving biological heterogeneity with a permuted surrogate variable analysis for genomics batch correction. Bioinformatics (2014) 30(19):2757–63. doi: 10.1093/bioinformatics/btu375 PMC4173013 24907368

[30] Uhlén M Fagerberg L Hallström BM Lindskog C Oksvold P Mardinoglu A. Proteomics. tissue-based map of the human proteome. Science (2015) 347(6220):1260419. doi: 10.1126/science.1260419 25613900

[31] Bateman A Martin MJ Orchard S Magrane M Agivetova R Ahmad S. UniProt: The universal protein knowledgebase in 2021. Nucleic Acids Res (2021) 49(D1):D480–9. doi: 10.1093/nar/gkaa1100 PMC7778908 33237286

[32] Chen T Zhang H Liu Y Liu YX Huang L. EVenn: Easy to create repeatable and editable Venn diagrams and Venn networks online. J Genet Genomics (2021) 48(9):863–6. doi: 10.1016/j.jgg.2021.07.007 34452851

[33] Szklarczyk D Gable AL Lyon D Junge A Wyder S Huerta-Cepas J. STRING v11: Protein-protein association networks with increased coverage, supporting functional discovery in genome-wide experimental datasets. Nucleic Acids Res (2019) 47(D1):D607–13. doi: 10.1093/nar/gky1131 PMC6323986 30476243

[34] Bader GD Hogue CW. An automated method for finding molecular complexes in large protein interaction networks. BMC Bioinf (2003) 4:2. doi: 10.1186/1471-2105-4-2 PMC149346 12525261

[35] Kumar R Indrayan A. Receiver operating characteristic (ROC) curve for medical researchers. Indian Pediatr (2011) 48(4):277–87. doi: 10.1007/s13312-011-0055-4 21532099

[36] Fava A Petri M. Systemic lupus erythematosus: Diagnosis and clinical management. J Autoimmun (2019) 96:1–13. doi: 10.1016/j.jaut.2018.11.001 30448290 PMC6310637

[37] Greloni G Scolnik M Marin J Lancioni E Quiroz C Zacariaz J. Value of repeat biopsy in lupus nephritis flares. Lupus Sci Med (2014) 1(1):e000004. doi: 10.1136/lupus-2013-000004 25396056 PMC4225737

[38] Nikitovic D Katonis P Tsatsakis A Karamanos NK Tzanakakis GN. Lumican, a small leucine-rich proteoglycan. IUBMB Life (2008) 60(12):818–23. doi: 10.1002/iub.131 18949819

[39] Kalamajski S Oldberg A. The role of small leucine-rich proteoglycans in collagen fibrillogenesis. Matrix Biol (2010) 29(4):248–53. doi: 10.1016/j.matbio.2010.01.001 20080181

[40] Chang PC Chen Y Lai MT Chang HY Huang CM Liu HP. Association analysis of polymorphisms in lumican gene and systemic lupus erythematosus in a Taiwan Chinese han population. J Rheumatol (2011) 38(11):2376–81. doi: 10.3899/jrheum.101310 21885486

[41] Wang X Hu J Fang Y Fu Y Liu B Zhang C. Multi-omics profiling to assess signaling changes upon VHL restoration and identify putative VHL substrates in clear cell renal cell carcinoma cell lines. Cells (2022) 11(3):472. doi: 10.3390/cells11030472 35159281 PMC8833913

[42] Gao S Yan L Zhang H Fan X Jiao X Shao F. Identification of a metastasis-associated gene signature of clear cell renal cell carcinoma. Front Genet (2020) 11:603455. doi: 10.3389/fgene.2020.603455 33613617 PMC7889952

[43] Wu J Lin Q Li S Shao X Zhu X Zhang M. Periostin contributes to immunoglobulin a nephropathy by promoting the proliferation of mesangial cells: A weighted gene correlation network analysis. Front Genet (2020) 11:595757. doi: 10.3389/fgene.2020.595757 33488671 PMC7817997

[44] Wright RD Dimou P Northey SJ Beresford MW. Mesangial cells are key contributors to the fibrotic damage seen in the lupus nephritis glomerulus. J Inflammation (Lond) (2019) 16:22. doi: 10.1186/s12950-019-0227-x PMC6857320 31807119

[45] Zhao X Hao J Duan H Rong Z Li F. Phosphoinositide 3-kinase/protein kinase b/periostin mediated platelet-derived growth factor-induced cell proliferation and extracellular matrix production in lupus nephritis. Exp Biol Med (Maywood) (2017) 242(2):160–8. doi: 10.1177/1535370216668050 PMC5167113 27590500

[46] Wantanasiri P Satirapoj B Charoenpitakchai M Aramwit P. Periostin: a novel tissue biomarker correlates with chronicity index and renal function in lupus nephritis patients. Lupus (2015) 24(8):835–45. doi: 10.1177/0961203314566634 25593049

[47] Zoshima T Baba T Tanabe Y Ishida Y Nakatani K Nagata M. CCR2- and CCR5-mediated macrophage infiltration contributes to glomerular endocapillary hypercellularity in antibody-induced lupus nephritis. Rheumatol (Oxford) (2021) 61(7):3033–48. doi: 10.1093/rheumatology/keab825 34747459

[48] Zeng Y Zhang Y Lin Y Wang X Chen Q Huang Q. The CXCL13 chemokine serves as a potential biomarker to diagnose systemic lupus erythematosus with disease activity. Clin Exp Med (2021) 21(4):611–9. doi: 10.1007/s10238-021-00707-x 33844093

